# Management of a complex glomus jugulare tumor with severe brainstem compression

**DOI:** 10.3171/2019.10.FocusVid.19461

**Published:** 2019-10-01

**Authors:** Diego Mendez-Rosito, Iván Méndez Guerrero, Sergio Reyna Heredia, David Gallardo Ceja

**Affiliations:** 1 Skull Base Program,; 2 Department of Neurological Surgery, Centro Médico Nacional 20 de Noviembre, ISSSTE, Mexico City, Mexico

**Keywords:** glomus jugulare, brainstem compression, jugular foramen, mastoidectomy, video

## Abstract

Surgery of glomus jugulare tumors is complex, but when these tumors have a large intradural component and cause brainstem compression they became a challenge to the surgeon. It is crucial to preoperatively understand the anatomy of the tumor and analyze its relationship with the temporal bone, facial nerve, and foramen jugular neurovascular structures. We present a case of a male patient with a complex glomus jugulare tumor causing severe brainstem compression. Nuances of technique and surgical pearls related to the management of complex jugulare tumors are discussed and illustrated in this operative video.

The video can be found here: https://youtu.be/oCjzp0kFRGc.

## Transcript

My name is Diego Méndez-Rosito from Centro Médico Nacional 20 de Noviembre in Mexico City and we are presenting the video titled “Management of a Complex Glomus Jugulare Tumor with Severe Brainstem Compression.”

This is the case of a 61-year-old male patient, who began 2 years prior to consultation with facial nerve palsy and hoarseness. Three months prior to admission he presented gait unsteadiness which worsened until severe inability to mobilize out of bed independently.

The neurological examination showed IV to XII right cranial nerve deficit and signs of brainstem and cerebellar compression.

A preoperative MRI showed a large tumor which enhanced with gadolinium, with an extra- and intracranial component that extended and eroded the jugular foramen. The intracranial extension had a large intradural component causing important brainstem compression in the cerebellopontine angle up to the perimesencephalic cisterns. The tumor is invading the posterior cavernous sinus and indenting above the tentorium.

A preoperative cerebral angiogram was done showing the important vascularity due to the ascending pharyngeal artery and external carotid branches. The right and left ICA also contribute with the vascular supply of the tumor through the meningohypophyseal trunk in its cavernous segment as well as through the vertebrobasilar system showing the vascular blush shown here.

A 3D reconstruction was done with a CT angiogram showing the large intracranial component of the tumor with bone erosion of the skull base. A preoperative 3D simulation of the surgical approach was done to show the extracranial and the expected intradural component of the tumor.

Now, considering the severe mass effect to the brainstem and the nourished vasculature of the tumor from external and internal carotid artery as well from the vertebrobasilar system, a preoperative embolization was done 48 hours prior to the surgery. At this point it’s important to set the goal of the surgery, which is to remove the highlighted lesion to decompress the brainstem (Al-Mefty and Teixeira, 2002).

The patient was fixed in a Mayfield and the head was rotated to the contralateral side. A retroauricular incision was planned, extending it down to the sternocleidomastoid muscle.

The superficial layers are reflected anteriorly while the sternocleidomastoid muscle is disinserted and reflected posteriorly, making sure to preserve the greater auricular nerve in case a graft is needed. It is important to plan a temporalis muscle superficial fascia for reconstruction.

The mastoid process is exposed while the upper cervical region is dissected, emphasizing in the muscle and neurovascular anatomy. The posterior belly of the digastric muscle is sectioned to expose the higher jugular vein, which together with the internal carotid artery will have intimate relationship with the vagus nerve. At the level of the angle of the mandible the hypoglossal nerve is positioned anteriorly to reach the muscles of the tongue. The branches of the external carotid artery are identified and ligated, the superior thyroid artery, the ascending pharyngeal artery, lingual artery, and facial artery, posteriorly the occipital artery.

After we have vascular control, we proceed to do the mastoidectomy; we like to harvest the cortical mastoid bone for reconstruction. When the mastoid cells are exposed, we use diamond drilling until exposing the duramater in the presigmoid Troutman triangle. The tumor is now exposed. The sigmoid sinus is eggshelled.

As it can be seen, the tumor is aggressively bloody even though we diminished the arterial vascular supply from the external carotid artery branches. After the tailored mastoidectomy is done, a retrosigmoid craniotomy is done exposing the craniocervical junction. The lateral mass of C1 and the vertebral artery are exposed.

Now the posterior fossa duramater is opened, prior to the placement of an external ventricular drainage to the atrium. The tumor is shown in the CPA angle. The tumor is still congestive, so we proceed to ligate the sigmoid sinus below the junction of the superior petrosal sinus, as it is shown here. After it is ligated, we can start remodeling and dissecting the tumor with bipolar coagulation. CSF is obtained from the cisterna magna to decompress. The dissection of the tumor kept on going with bipolar coagulation.

Once a wide exposure of the tumor is obtained, we can see at this point that we have a very solid tumor. We cut it using an 11 blade and we try to suction and coagulate. We find that using microscissors we can delimit better blocks of tumor together with coagulation, as it is shown here. The debulking is done with sectioning and coagulation of the tumor.

At this point the ligated sigmoid sinus is sectioned to do a transsinus approach to have better exposure of the tumor extending to the crural cistern. The tentorium is sharply sectioned until identifying the free edge and the fourth cranial nerve as it is shown here. We continue with the tumor debulking.

Now, the jugular vein is ligated to trap the tumor proximally and distally. At this point the jugular vein can be opened to find the residual, the lower segment of the tumor, where this tumor is going to be followed upward to the jugular bulb, always preserving the medial wall of the vein (Borba et al., 2010).

Now we can see the brainstem decompression and the identification of the fourth cranial nerve, the trigeminal nerve, and the VII–VIII complex.

The reconstruction was done with suture and dural substitute, fascia lata, and the vascularized pedicle from temporalis muscle, with fibrin glue. The external ventricular drainage is kept in the immediate postop (Candido et al., 2019).

A postoperative MRI shows a subtotal resection showing an important brainstem decompression, leaving the extradural component of the tumor (De Brito et al., 2018). The residual was treated with radiotherapy (Sahyouni et al., 2018; Shapiro et al., 2018).

The postop the patient improved the gait and the brainstem compressive symptoms. The cranial nerve deficit remained unchanged as in the preop.

## Time points

0:35 Clinical presentation
1:04 Preoperative MRI
1:35 Preoperative cerebral angiogram
2:23 Discussion
2:50 Positioning
3:02 Surgery
8:41 Postop MRI
8:57 Follow-up

## Disclosures

The authors report no conflict of interest concerning the materials or methods used in this study or the findings specified in this publication.
